# A Priori Design of Dual-Atom Alloy Sites and Experimental Demonstration of Ethanol Dehydrogenation and Dehydration on PtCrAg

**DOI:** 10.1021/jacs.2c13577

**Published:** 2023-03-08

**Authors:** Paul L. Kress, Shengjie Zhang, Yicheng Wang, Volkan Çınar, Cynthia M. Friend, E. Charles H. Sykes, Matthew M. Montemore

**Affiliations:** † Department of Chemistry, Tufts University, Medford, Massachusetts 02155, United States; ‡ Department of Chemical and Biomolecular Engineering, Tulane University, New Orleans, Louisiana 70118, United States; § Department of Chemistry and Chemical Biology, Harvard University, Cambridge, Massachusetts 02138, United States

## Abstract

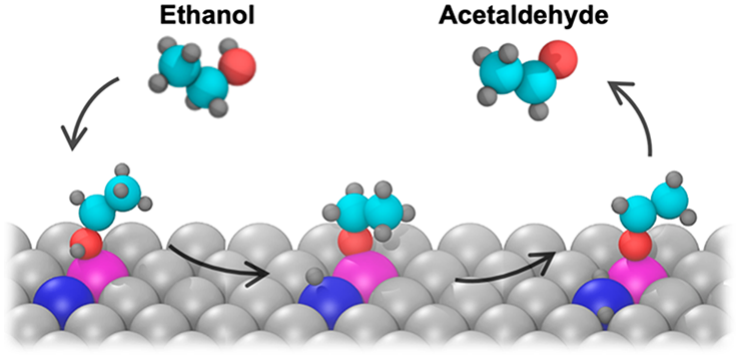

Single-atom catalysts have received significant attention for their ability to enable highly selective reactions. However, many reactions require more than one adjacent site to align reactants or break specific bonds. For example, breaking a C–O or O–H bond may be facilitated by a dual site containing an oxophilic element and a carbophilic or “hydrogenphilic” element that binds each molecular fragment. However, design of stable and well-defined dual-atom sites with desirable reactivity is difficult due to the complexity of multicomponent catalytic surfaces. Here, we describe a new type of dual-atom system, trimetallic dual-atom alloys, which were designed via computation of the alloying energetics. Through a broad computational screening we discovered that Pt–Cr dimers embedded in Ag(111) can be formed by virtue of the negative mixing enthalpy of Pt and Cr in Ag and the favorable interaction between Pt and Cr. These dual-atom alloy sites were then realized experimentally through surface science experiments that enabled the active sites to be imaged and their reactivity related to their atomic-scale structure. Specifically, Pt–Cr sites in Ag(111) can convert ethanol, whereas PtAg and CrAg are unreactive toward ethanol. Calculations show that the oxophilic Cr atom and the hydrogenphilic Pt atom act synergistically to break the O–H bond. Furthermore, ensembles with more than one Cr atom, present at higher dopant loadings, produce ethylene. Our calculations have identified many other thermodynamically favorable dual-atom alloy sites, and hence this work highlights a new class of materials that should offer new and useful chemical reactivity beyond the single-atom paradigm.

## Introduction

The design of well-defined active sites in heterogeneous catalysis is a long-standing goal of the field, but the structural complexity of supported catalysts and their dynamic changes in response to reactive atmospheres complicate this goal.^1,2^ The advent of single-atom, or more precisely, single-site, catalysis has offered an approach to localizing the active site to a single metal atom coordinated to the support, typically an oxide.^3,4^ Supported single-atom catalysts can have high performance but are often structurally complex. Furthermore, preventing aggregation of the single atoms in these systems can be challenging. Most recently, dual-atom sites have been identified as promising active sites for several reactions.^5−10^ However, supported dual-atom catalysts are again structurally complex, making them difficult to design, synthesize, and characterize.^11^ This complexity also impedes understanding the reaction mechanism at a fundamental level. While computational screening of these materials can identify candidates with high predicted performance,^11^ synthesizing and characterizing the predicted structures can be difficult in many cases.

As an alternative to oxide-supported dual-atom catalysts, it may be possible to create stable heterometallic pairs embedded in a third metal host.^12,13^ These dual-atom alloys would feature active sites that are amenable to atomic-scale characterization with scanning tunneling microscopy (STM) and accurate modeling with theory. Furthermore, unlike supported single-atom and dual-atom catalysts where the dopant is often charged, metal dopants in metal supports have near 0 valence.^14^ The dual-atom alloy structure is analogous to single-atom alloys (SAAs), which consist of catalytically active metal atoms atomically dispersed in a second, typically more inert, host metal. However, dual-atom alloys extend beyond SAAs, as they feature a two-atom active site, which is a significantly larger and more tunable active site than that of SAAs. SAAs have proven to be effective catalysts for many reactions,^15−21^ and their well-defined active sites enable them to be highly selective and amenable to first-principles design.^22^ The site isolation in SAAs also provides important catalytic benefits that may be shared by dual-atom alloys, such as enabling spillover of reaction intermediates and deviations from linear scaling relationships that limit the performance of traditional catalysts.^15,16,23−25^ However, single-atom active sites may not be able to stabilize larger intermediates or transition states, or break very strong bonds; therefore, more challenging reactions will require adjacent active atoms.^26,27^

Dual-atom alloys address these limitations of SAAs by combining two active atoms with different chemical reactivity in one localized heteroatom dimer active site. A dimer site comprised of two different dopant metals should allow much more tunability of reactant binding and activation and hence improved control over chemical reactivity beyond what can be achieved with SAAs. However, due to the enormous structure space involved with the design of trimetallic alloy sites, effective computational screening is crucial for a priori identification of the most stable and chemically reactive combinations.

In this work, we computationally design and experimentally synthesize and test a dual-atom alloy consisting of a heterometallic dimer, Pt–Cr, embedded in an inert host, Ag. The use of a Ag(111) single-crystal host enabled us to directly image the different active sites and demonstrate that Pt–Cr dimer sites can dehydrogenate ethanol in temperature-programmed desorption (TPD) studies, while both Pt_1_Ag and Cr_1_Ag are inert for this reaction. Our calculations on the reaction mechanism reveal that adjacent oxophilic Cr and carbophilic or “hydrogenphilic” Pt atoms are necessary to provide favorable binding and reaction energies for the conversion of ethanol to acetaldehyde and that higher dopant loadings lead to the formation of ethylene.

## Results and Discussion

**Figure 1:**
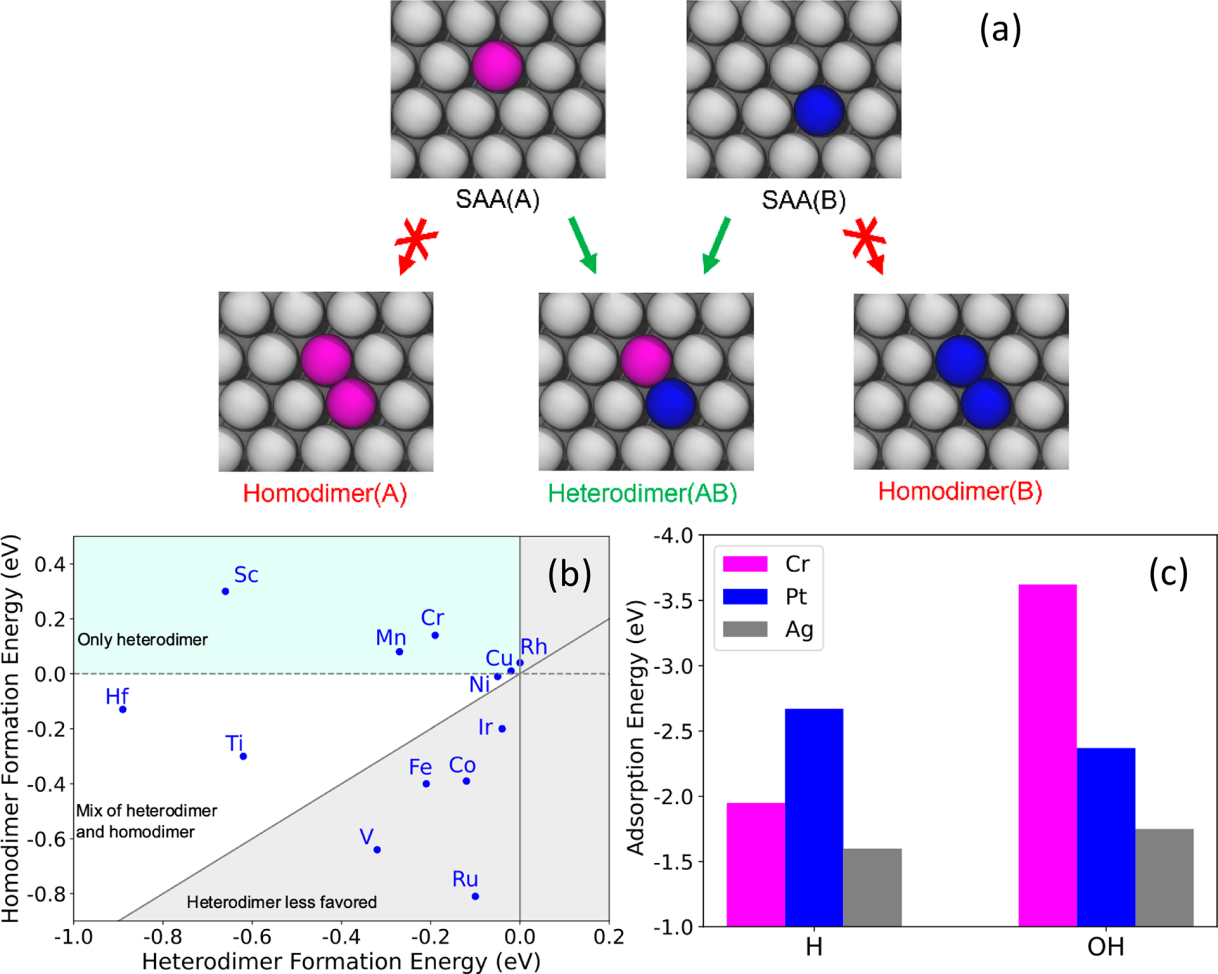
Identification of stable heteroatom dual-atom alloys. (a) Schematic showing the geometric alloy configurations that were computed to assess the stability of the heteroatom dual-atom pairs. (b) Computational screening of the stability of various heteroatom dimer sites based on the addition of a third metal to the Pt_1_Ag SAA. The homodimer and heterodimer formation energies are shown, and the upper left quadrant of the plot (shaded in light blue) shows the most promising candidates, which have negative heterodimer formation energies and positive homodimer energies. (c) DFT-calculated top-site H and OH adsorption energies on the PtCr-Ag dual-atom alloy showing the oxophilic nature of the Cr site and the “hydrogenphilic” nature of the Pt site.

In the search for synthesizable dual-atom alloy materials, we first computationally screened many alloy combinations in order to identify cases where the two different dopant atoms would form a heterometallic dimer as opposed to homometallic dimers (Figure 1a).^12^ Specifically, we started with the known Pt_1_Ag, Pd_1_Ag, Pd_1_Au, and Ti_1_Cu SAAs, which all have positive homometallic dimer formation energies and hence are stable SAAs. We used density functional theory (DFT) to calculate the heterometallic dimer formation energy for a wide range of elements as possible third metal dopants to add to these bimetallic SAA systems. These heterometallic dimer formation energies must be negative in order to favor formation of the heteroatom dimer site (Figure 1b, Figure S1). We also calculated the homometallic dimer formation energy of the third metal, which must be less negative than the heterometallic dimer so that the heterometallic dimer is energetically favored over the homodimer. From these screening results, many heteroatom dual-atom alloy sites are predicted to be stable, and the most promising in terms of active site uniformity are those with a negative heterometallic dimer formation energy but a positive homometallic dimer formation energy seen in the light blue regions of Figures 1b and S1.

To identify heteroatom dimer sites with desirable chemical reactivity for O–H activation, the first step of ethanol dehydrogenation, we searched for thermodynamically stable pairs in which one metal atom stabilizes an O-containing intermediate and the other metal atom binds H strongly. Based on previous work,^28^ Cr is relatively oxophilic, while Pt is relatively carbophilic, which suggests it will bind H strongly.^29^ Our DFT calculations of H and OH adsorbed on the top sites of the Pt_1_Cr_1_Ag dual-atom alloy confirmed this fact and demonstrate that the Pt atom of the dual site binds H more strongly than OH, while the opposite is true for the Cr atom, as seen in Figure 1c. In addition to its complementary reactivity to Pt, Cr was chosen over other promising candidates (Sc, Mn, and Cu) for several reasons. Cr has particularly desirable energetics for heterodimer formation, whereas Cu has a weak tendency to form heterodimers. Sc has a very negative bulk oxidation energy, which may lead to formation of large oxide clusters under some conditions, and can be difficult to work with experimentally. Furthermore, Cr is a common catalytic metal in industry, making understanding of its single-atom and dimer-pair behavior particularly important. Therefore, the Pt_1_Cr_1_Ag dual-atom alloy is predicted to be both thermodynamically stable and composed of two atoms with complementary chemical reactivity.

**Figure 2:**
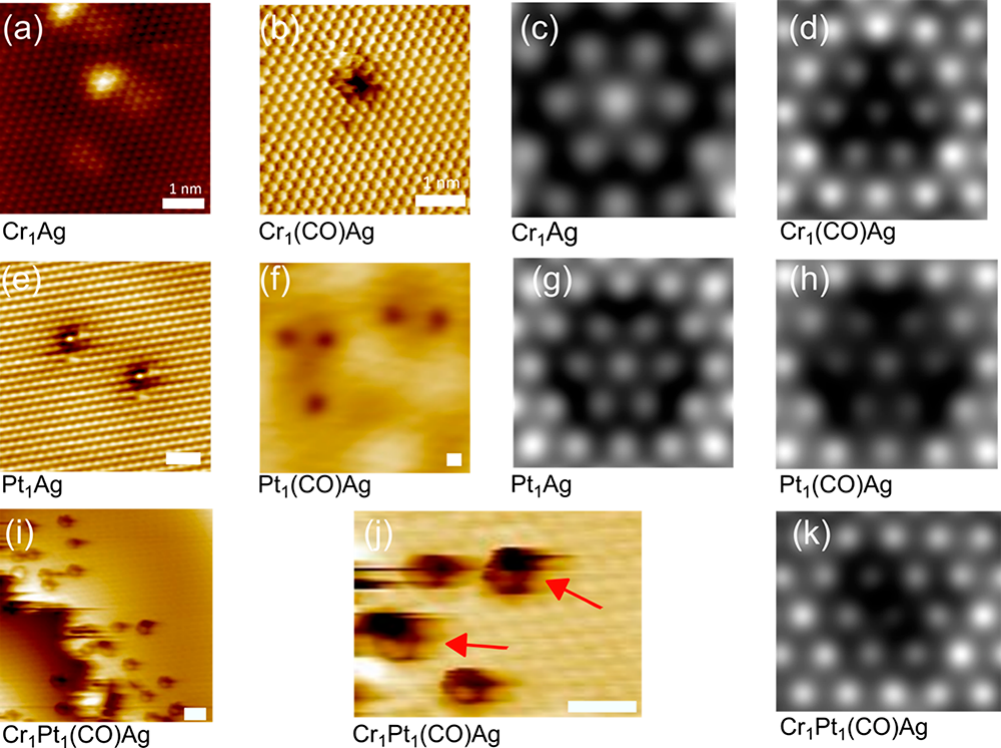
STM images of 1% of a monolayer CrAg, PtAg, and 0.5% CrPtAg alloy surfaces. (a) STM image of an isolated Cr atom in Ag(111). (b) STM image of CO bound to an isolated Cr atom. (c) Simulated STM image of a Cr atom in Ag without CO and (d) with CO. (e) STM image of isolated Pt atoms in Ag(111) without CO and (f) with CO adsorbed. (g) Simulated STM image of a Pt atom in Ag without CO and (h) with CO. (i) Zoomed-out STM image of the CrPtAg alloy in the region around a step edge. (j) Atomic resolution of PtCrAg alloy showing two heterodimer sites highlighted with red arrows and two isolated Pt atom sites. (k) Simulated STM image of the heteroatom PtCr site with CO. All scale bars are 1 nm.

Based on this computational screening that identified the Pt_1_Cr_1_Ag dual-atom alloy as having favorable thermodynamic stability and desirable chemical properties for ethanol activation, we synthesized and characterized the trimetallic PtCrAg alloy and its PtAg and CrAg bimetallic counterparts. First, Pt was deposited onto a clean Ag(111) single crystal via electron beam evaporation at an alloying temperature of 380 K. This led to the formation of a PtAg SAA^30^ in which the Pt atoms are dispersed in the Ag surface in the form of isolated atoms as seen in Figures 2e,f and S3. These STM images show the appearance of the single-atom Pt sites with and without adsorbed CO, which is present in the ultra-high-vacuum (UHV) chamber background. Next, the alloying of Cr with Ag(111) was investigated. Given that our goal is to form heteroatom pairs, we used Cr(CO)_6_ in the gas phase to add the Cr. Our hypothesis was that Cr(CO)_6_ is less reactive than evaporated Cr metal atoms, and hence the longer surface residence times before alloying enable the Cr(CO)_6_ to sample different surface sites and decompose next to the Pt atom sites. Specifically, Cr(CO)_6_ should be more likely to decompose at Pt sites which bind CO strongly as opposed to Ag sites that bind CO weakly; hence Pt should facilitate the decomposition of the carbonyl. As we will describe below, this kinetic trapping of the Cr atom next to the Pt site, combined with the intrinsic thermodynamic stability of the PtCr dimer pair confirmed by our computational studies, provides a new method for synthesizing these dual-atom sites.

Figures 2a,b and S2 show the results of alloying Cr into Ag(111) by exposure to Cr(CO)_6_ at a sample temperature of 320 K. Just as with Pt, both bright and dark protrusions are observed, and the comparison to the DFT-simulated STM images shown in panels c and d reveals that CO-free Cr atoms appear as protrusions and Cr atoms with adsorbed CO appear as depressions. Most interestingly, when both Pt and Cr are alloyed with Ag(111), in addition to isolated Pt and Cr atoms, we observe the appearance of oblong asymmetric features aligned with the close-packed directions of the Ag(111) surface (Figures 2j, S4, and S5). These STM images, coupled with the DFT-simulated images, indicate that Pt–Cr heteroatom dimers are formed via the serial deposition of Pt and Cr(CO)_6_ on Ag(111).

**Figure 3:**
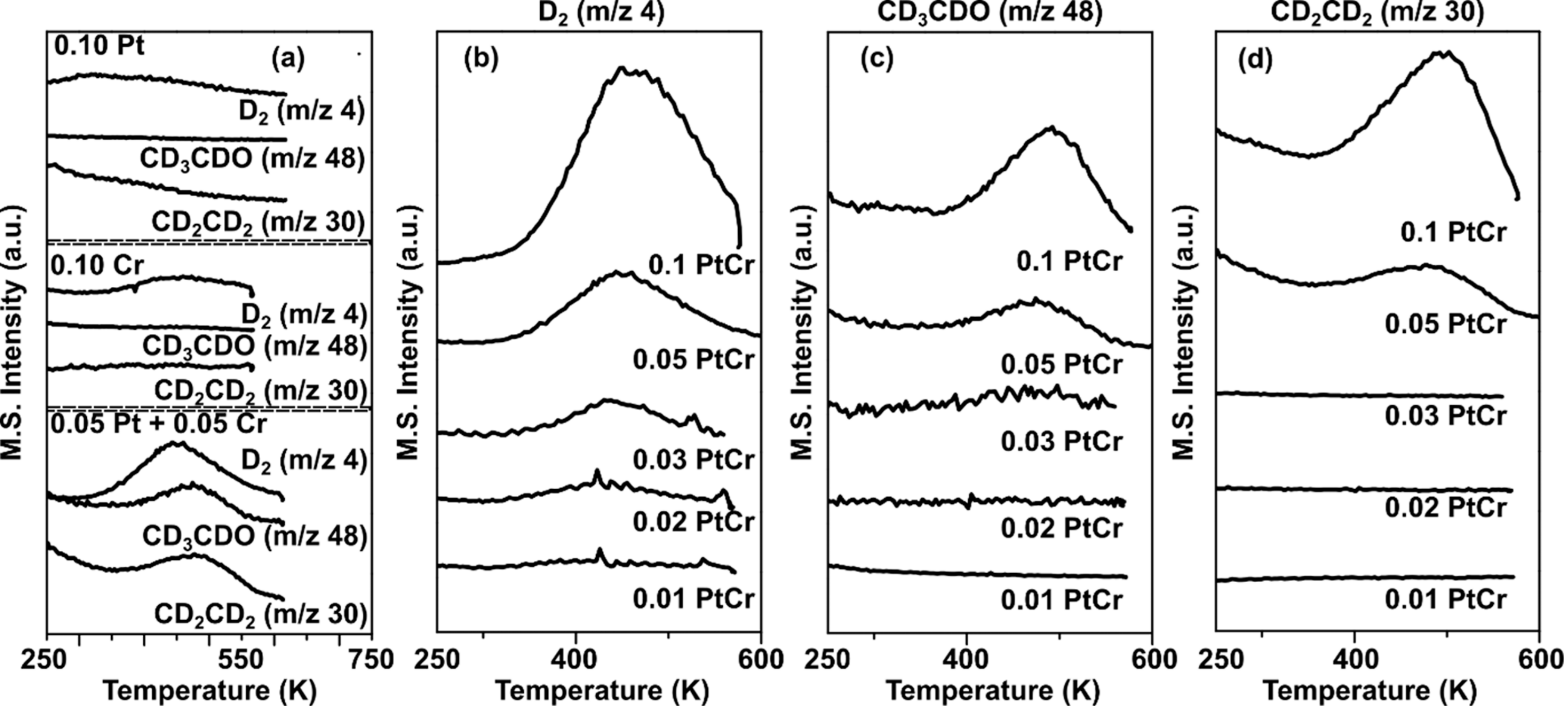
Product yields from the reaction of ethanol on CrAg, PtAg, and PtCrAg surface alloys. Panel a shows that the bimetallic CrAg and PtAg alloys with 10% of the dopant are inactive for ethanol decomposition, as seen by the lack of products desorbing vs the production of acetaldehyde, D_2_, and ethylene from the 5% Pt and 5% Cr in the Ag(111) trimetallic surface. Panels b–d show evolution of these products over a range of dopant coverages (in monolayers) of the trimetallic PtCrAg alloy.

**Figure 4:**
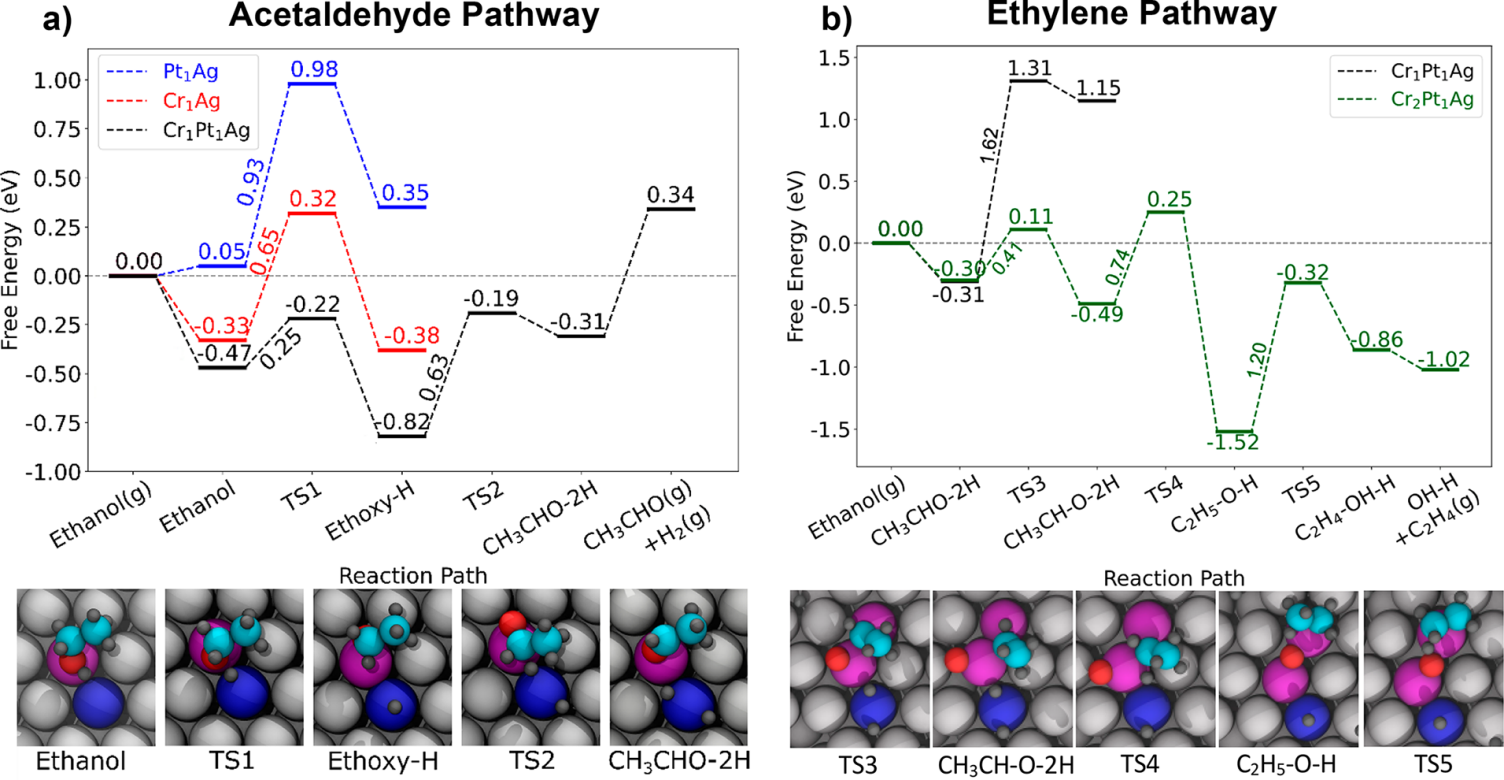
DFT-calculated energetics of (a) ethanol dehydrogenation to acetaldehyde on Pt_1_Ag, Cr_1_Ag, and the Pt_1_Cr_1_Ag dual-atom alloy and (b) further reaction to ethylene on Pt_1_Cr_1_Ag and Pt_1_Cr_2_Ag. The Pt_1_Cr_1_ dimer site kinetically and thermodynamically facilitates initial O–H bond breaking significantly more than the respective SAAs, and the Pt_1_Cr_2_ trimer site facilitates C–O bond cleavage. Images of intermediates and transition states along the pathways are shown. Free energies are calculated at 300 K, 1 atm.

After characterizing the atomic-scale structures of the PtAg, CrAg, and PtCrAg alloys, we tested their reactivity for ethanol decomposition to acetaldehyde, hydrogen, and ethylene using TPD experiments. This reaction was chosen as a simple but important example where O–H bond breaking is critical, but complete decomposition of the molecule is undesirable.^31−35^ Our goal was not necessarily to design a better catalyst for this reaction, as there are existing alloys that can efficiently convert ethanol.^32,33^ TPD studies do not directly probe catalytic turnover, as the experiment involves a single deposition of ethanol followed by heating of the sample to induce reaction. Instead, we use this reaction as a probe of the reactivity of the Pt–Cr heterodimer sites and compare them to Pt and Cr single-atom sites. In order to more accurately detect the desorption of reactively formed hydrogen, we exposed the surface to fully deuterated ethanol and detected deuterated acetaldehyde and D_2_ as products. As seen in Figures 3a, S6, and S7, the PtAg and CrAg bimetallic surfaces do not decompose ethanol even at loadings up to 10% of a monolayer. The PtAg results are in agreement with previous TPD studies of ethanol on Pt(111), which observe no reaction, merely reversible adsorption/desorption of intact ethanol.^36^ In contrast, the presence of just 3% of both Cr and Pt results in activity toward ethanol dehydrogenation as seen in Figure 3b–d. This indicates that the dual-atom Pt–Cr dimer site in Ag is capable of performing a reaction that does not occur on either Pt or Cr single-atom sites. Figure S7 shows full TPD data for all alloy compositions studied. Interestingly, at low dopant concentrations, ∼3% of a monolayer, the mixed PtCrAg sample is 100% selective to acetaldehyde and hydrogen, and as the concentration of the dopants is increased, the selectivity to ethylene increases, reaching ∼80% at 10% dopant concentrations (see Table S1). Overall, the TPD results indicate that varying the dopant loading modifies the observed reaction products:
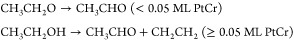


Our DFT calculations rationalize these results and show that the different reactivities of the two atoms of the Pt_1_Cr_1_ dual-atom alloy site are crucial for O–H bond activation in ethanol (Figures 4a and S8, and Table S2). Specifically, in the dissociated ethoxy+H state, Pt stabilizes the H atom, as it is quite hydrogenphilic. However, on Pt_1_Ag, ethoxy is forced to a Ag site, where it is relatively unstable. Conversely, Cr is oxophilic, such that ethoxy is quite stable on Cr_1_Ag, but H is forced to a Ag site, where it is relatively unstable. Hence, there is little or no thermodynamic driving force for ethanol to dissociate on either of the respective SAAs as seen in Figure 4. However, in the mixed dimer, Cr stabilizes ethoxy while Pt stabilizes H, thus lowering the energy of both the final state and the transition state and creating a favorable energetic landscape for the complete reaction of ethanol to acetaldehyde and hydrogen as seen in Figure 4a. This contrasts with the PtAg and CrAg SAAs, on which it can be seen that desorption of ethanol is kinetically favored over breaking the O–H bond, consistent with our TPD results in which ethanol desorbed intact from both bimetallic surfaces. Furthermore, we find that adding a second Cr atom to form a larger ensemble significantly changes the reaction energetics to favor C–O cleavage, making ethylene formation more energetically favorable, as seen in Figures 4b and S9–S11. This indicates that larger Pt–Cr_*n*_ ensembles are the active sites for ethylene production. Specifically, on the Pt–Cr dimer, the C–O bond scission was calculated to have a high barrier of 1.62 eV, while the Pt–Cr_2_ trimer gives a much lower calculated barrier, 0.41 eV. On the trimer, the C–O bond scission transition state features the O interacting with a Cr, the ethyl fragment interacting with the other Cr, and both H atoms adsorbed on the Pt. Furthermore, the calculated formation energies of two types of trimers are both negative: −0.12 eV for Cr_2_Pt_1_Ag forming from Cr_1_Pt_1_Ag and Cr_1_Ag and −0.15 eV for Cr_1_Pt_2_Ag forming from Cr_1_Pt_1_Ag and Pt_1_Ag. This result suggests that the trimers are likely to form in the surface as the concentration of Cr and Pt increases. This is consistent with the experimental findings that ethylene production dominates the reaction selectivity at higher coverages of Pt and Cr.

## Conclusion

In summary, we have computationally designed and experimentally tested a new type of active site, the dual-atom alloy, in which heterodimers of two reactive atoms (Pt and Cr) are embedded in a more inert host (Ag). This trimetallic was identified based on computational examination of both the stability and reactivity of the dual site. STM confirmed the presence of Pt–Cr sites in the experimentally synthesized trimetallic, and TPD demonstrated that these dual sites are active for ethanol dehydrogenation, while the individual component PtAg and CrAg bimetallics are not. DFT calculations revealed that the PtCr mixed site is needed in order for O–H bond breaking to be both thermodynamically and kinetically favorable, as Pt binds the dissociated H while Cr stabilizes the ethoxy intermediate. Moreover, the larger PtCr ensembles present at higher dopant loadings changed the reaction selectivity to favor the production of ethylene, demonstrating how sensitive the reaction pathway is to the atomic-scale structure of the heteroatom active site. More generally, this work opens up a new class of dual-atom alloy materials that can be computationally designed using the methods we developed here. These materials extend beyond SAAs to a larger design space and larger active site. The well-defined nature of the dual-atom active site provides a strong synergy between computational modeling and experiment, which is critical in the quest to design catalytic sites from first principles. For example, this approach involving fundamental studies of the active site structure and associated reactivity has proven very successful in the recent development of a variety of SAA catalysts.^15,24,37^ Our DFT screening highlights many other dual-atom alloy materials that are predicted to be stable and hence synthesizable. Compared to the relatively small structure space of SAAs, these dual-atom alloys offer many stable and potentially useful heteroatom active sites and feature a larger active site in which the two different elements can stabilize distinctly different chemical intermediates. These mixed-metal active sites also offer the intriguing possibility of performing selective cross-coupling reactions between two different species by virtue of the reactants’ preferred binding to each of the two different atoms of the dual site.

## Experimental Section

### Experimental Methods

The surface science experiments were performed on an Omicron low-temperature STM and a separate home-built TPD chamber. Both chambers have a base pressure < 1 × 10^–10^ mbar. The Ag(111) single crystal (99.999%, Princeton Scientific) was cleaned via Ar^+^ sputtering followed by 750 and 1000 K anneals in the TPD and STM chambers, respectively. The PtAg alloy was synthesized following a previously reported procedure, in which Pt was deposited using an electron beam evaporator onto the Ag(111) surface held at 380 K.^30^ The Cr(CO)_6_ was deposited using the vapor pressure above solid Cr(CO)_6_ purchased from Sigma-Aldrich (98% purity). The compound was placed into a glass sample vial and connected via a turbo-pumped gasline to a precision leak valve on the UHV chambers. The evacuated pressure in the gas line was <1 × 10^–4^ mbar, and once the Cr(CO)_6_ vial was opened to the gas line, the pressure rose to 5 × 10^–1^ mbar. During Cr(CO)_6_ deposition the Ag(111) crystal was held at 320 K, and the pressure in the chamber was 1 × 10^–6^ mbar during the deposition. Liquid ethanol-*d*_6_ (Sigma-Aldrich 99.5%) was purified via freeze–pump thaw cycles prior to use in order to remove any dissolved gases. Ethanol doses are given in Langmuirs (1 L = 1 × 10^–6^ Torr·s). The TPD experiments were collected with a heating rate of 1.5 K/s. Quantitative mass spectrometry was used to determine the surface coverage of the Pt and Cr dopants using CO TPDs following a literature procedure.^38^ The TPD traces in Figure 3 were background subtracted to remove the signal from the desorption of unreacted D-ethanol (*m*/*z* = 34) from the acetaldehyde (*m*/*z* = 48) signal. Product selectivity was calculated by correcting the desorption peaks for ionization cross section, fragmentation pattern, and the mass spectrometer quadrupole sensitivity.^39^ In the TPD experiments the surface temperature is ramped up to 600 K, which leads to the dopants diffusing beneath the surface, which is a common phenomenon in TPD studies of dilute alloys.

### Computational Methods

All periodic plane wave DFT calculations were carried out by using the Perdew–Burke–Ernzerhof generalized gradient (GGA-PBE)^40^ exchange–correlation functional with the Tkatchenko–Scheffler method^41^ for van der Waals interactions and were performed with the Vienna Ab initio Simulation Package (VASP).^42,43^ The cutoff energy for all the calculations was set to 400 eV. The surfaces calculated in this work are all fcc(111) structures, and the relaxation calculations were done on a 7 × 7 × 1 *k*-point mesh with 3 × 3 × 4 unit cells with the bottom two layers fixed. The lattice constants and the convergence tests were performed in our previous work.^44^ The transition states were optimized by the improved dimer method^45^ and were confirmed by frequency calculations. The harmonic approximation was used to calculate free energy corrections and CO vibrational frequencies (Table S3). For STM image simulation, a bias voltage of −200 mV was applied; Cr_1_Ag, Cr_1_(CO)Ag, Pt_1_Ag, and Pt_1_(CO)Ag structures were modeled with a 5 × 5 × 4 unit cell, and Cr_1_Pt_1_(CO)Ag was modeled with a 6 × 6 × 4 unit cell. Spin-polarized calculations with different initial magnetism were performed to determine the most stable spin state of each dopant–host system.

## Supporting Information Available

Additional STM images, additional screening calculations, additional TPD data, additional structures and energetics from DFT, calculated CO vibrational frequencies (PDF)
