# Supplementary material for: A Priori Design of Dual-Atom Alloy Sites and Experimental Demonstration of Ethanol Dehydrogenation and Dehydration on PtCrAg
Source: J Am Chem Soc. 2023 Mar 8;145(15):8401–7. doi: 10.1021/jacs.2c13577 (PMC10119928; doi:10.1021/jacs.2c13577)
Supplement: Supplementary file 1 — ja2c13577_si_001.pdf [file ja2c13577_si_001.pdf]

A priori design of dual-atom alloy sites and experimental demonstration of ethanol dehydrogenation and dehydration on PtCrAg

Paul L. Kress,[1,†] Shengjie Zhang,[2,†] Yicheng Wang,[1] Volkan Çinar,[1] Cynthia M. Friend,[3] E. Charles H. Sykes,[1,*] Matthew M. Montemore[2,**]

1 Department of Chemistry, Tufts University, Medford, MA 02155, USA

2 Department of Chemical and Biomolecular Engineering, Tulane University, New Orleans, LA 70118, USA

3 Department of Chemistry and Chemical Biology, Harvard University, Cambridge, MA 02138, USA

*charles.sykes@tufts.edu, **mmontemore@tulane.edu

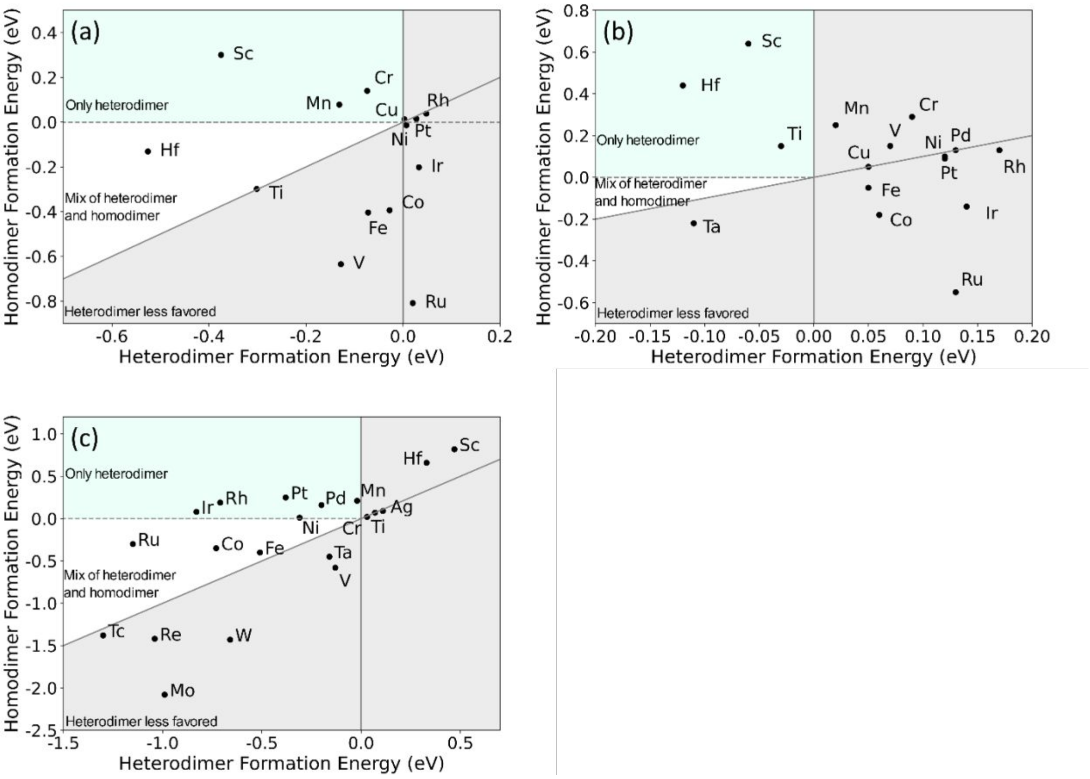

Figure S1 Computational screening of the stability of various dimers based on a (a)Pd_1Ag, (b)Pd_1Au and (c) Ti_1Cu SAAs as a host. The homodimer and heterodimer formation energies are shown.

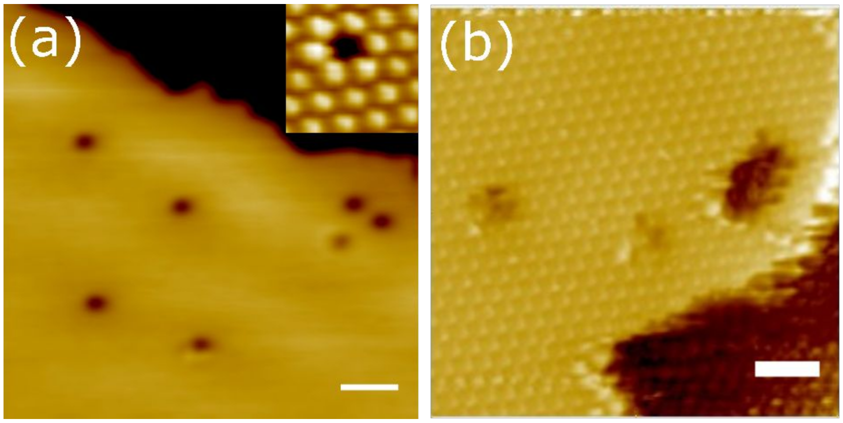

Figure S2 STM images of 1% CrAg(111) at 5 K a) Individual Cr single atoms embedded in the Ag(111) terrace above a step edge where individual Cr atoms appear as localized depressions. The inset shows atomic resolution of a Cr single atom in the Ag(111) surface b) In some areas of the surface Cr is also found in small islands which appear as depressions. Imaging condition: a) -100 mV, 0.6 nA inset -100 mV, 0.6 nA b) 100 mV, 0.7 nA. Scale bars: a) 2 nm b) 1 nm.

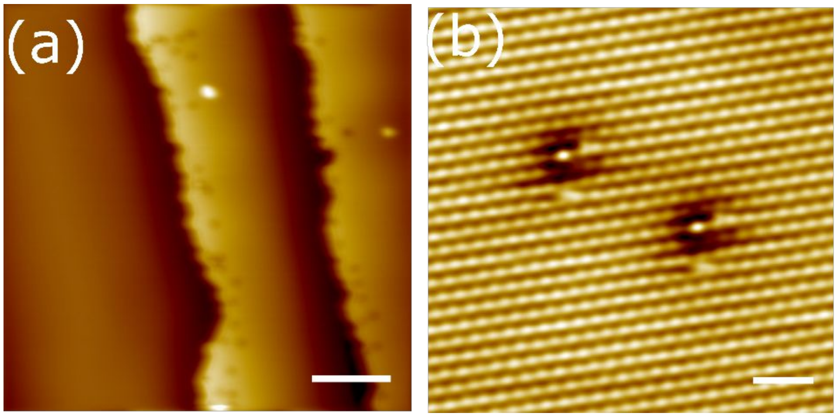

Figure S3 STM images of 1% PtAg(111) at 5 K a) Individual Pt single atoms appear in the Ag(111) terraces in the regions above step edges. Pt atoms appear as depressions. b) Atomically resolved images of two individual Pt atoms in the Ag(111) terraces which appear as shallow protrusions. Imaging condition: a) -50 mV, 1.5 nA b) 50 mV, 50 nA. Scale bars: a) 5nm b) 1 nm.

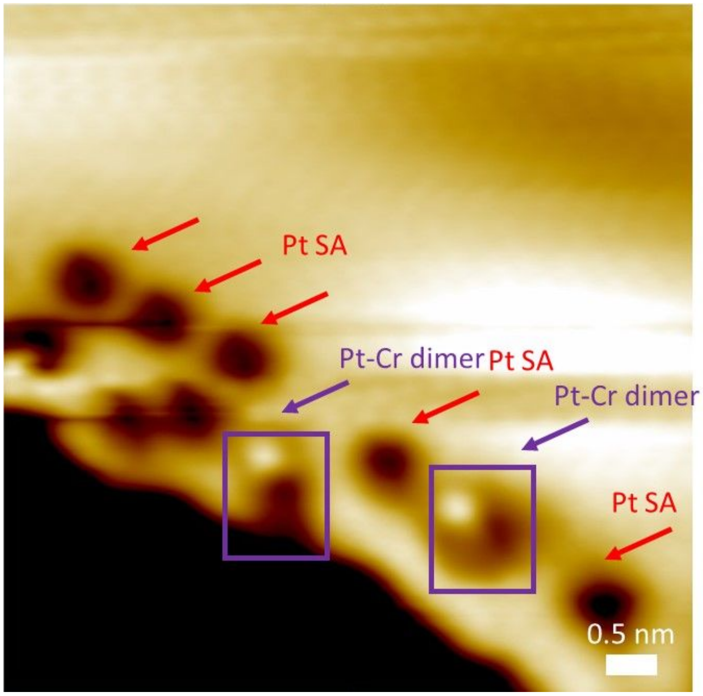

Figure S4. Higher resolution STM image of Pt-Cr dimer sites in 0.5% PtCrAg(111) acquired at 5 K. Pt atoms appear as depressions and Cr atoms appear as protrusions. Imaging condition: -10 mV, 40 nA.

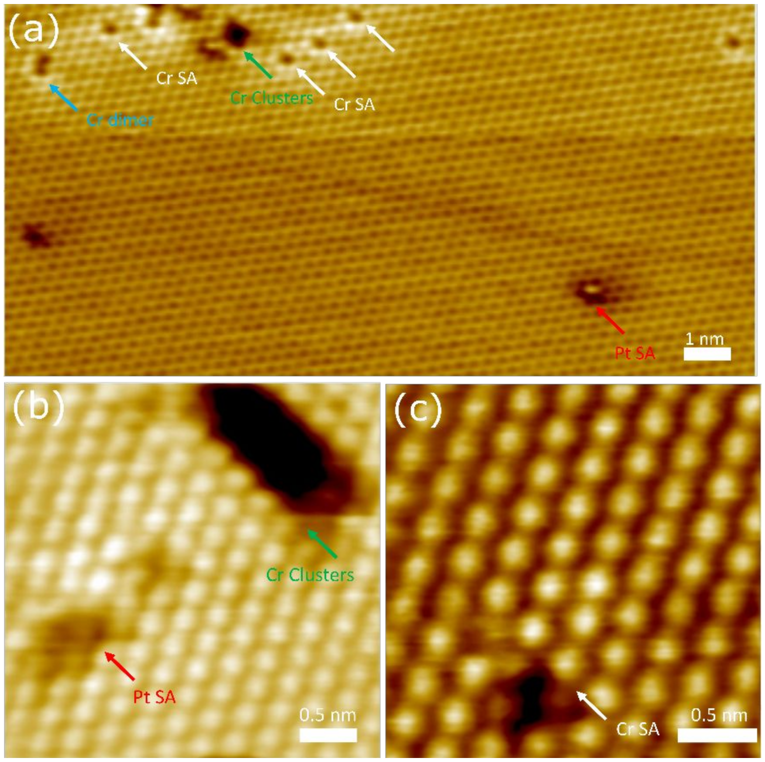

Figure S5. STM images of various Cr features in 0.5% PtCrAg(111) acquired at 5 K. a) Atomic resolution of mixed features of Cr single atoms, Cr dimers and Cr clusters as well as Pt single atoms. Cr appears as depressions while Pt appears as protrusions. b) Zoomed-in view of Cr clusters and Pt single atoms. c) Zoomed-in view of Cr single atoms. These STM images demonstrate that in addition to single atoms, when Cr is alloyed into Ag(111) via a carbonyl, it tends to form mixed features of single atoms, dimers and clusters that TPD studies show are inactive towards ethanol dehydrogenation or dehydration. Imaging conditions: a) -120 mV, 0.4 nA. b) and c) -150 mV, 0.4 nA.

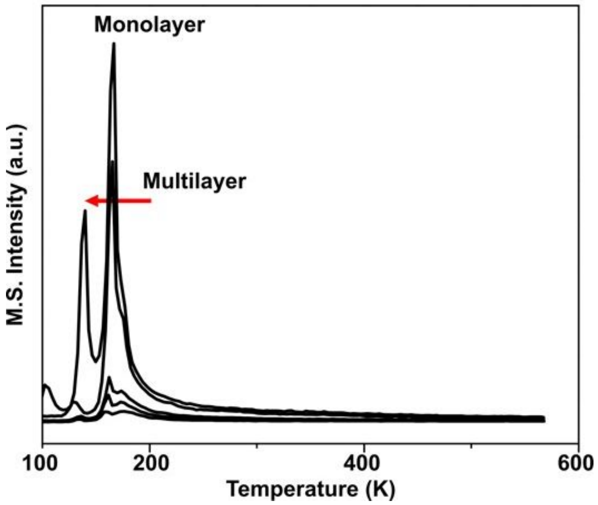

Figure S6 TPD traces for the desorption of 0.2 L, 0.5 L, 1 L, 4 L and 8 L ethanol (CH_3CH_2OH) from Ag(111) following m/z = 29. The monolayer peak saturates ~4 L and a multilayer peak ~130 K is observed.

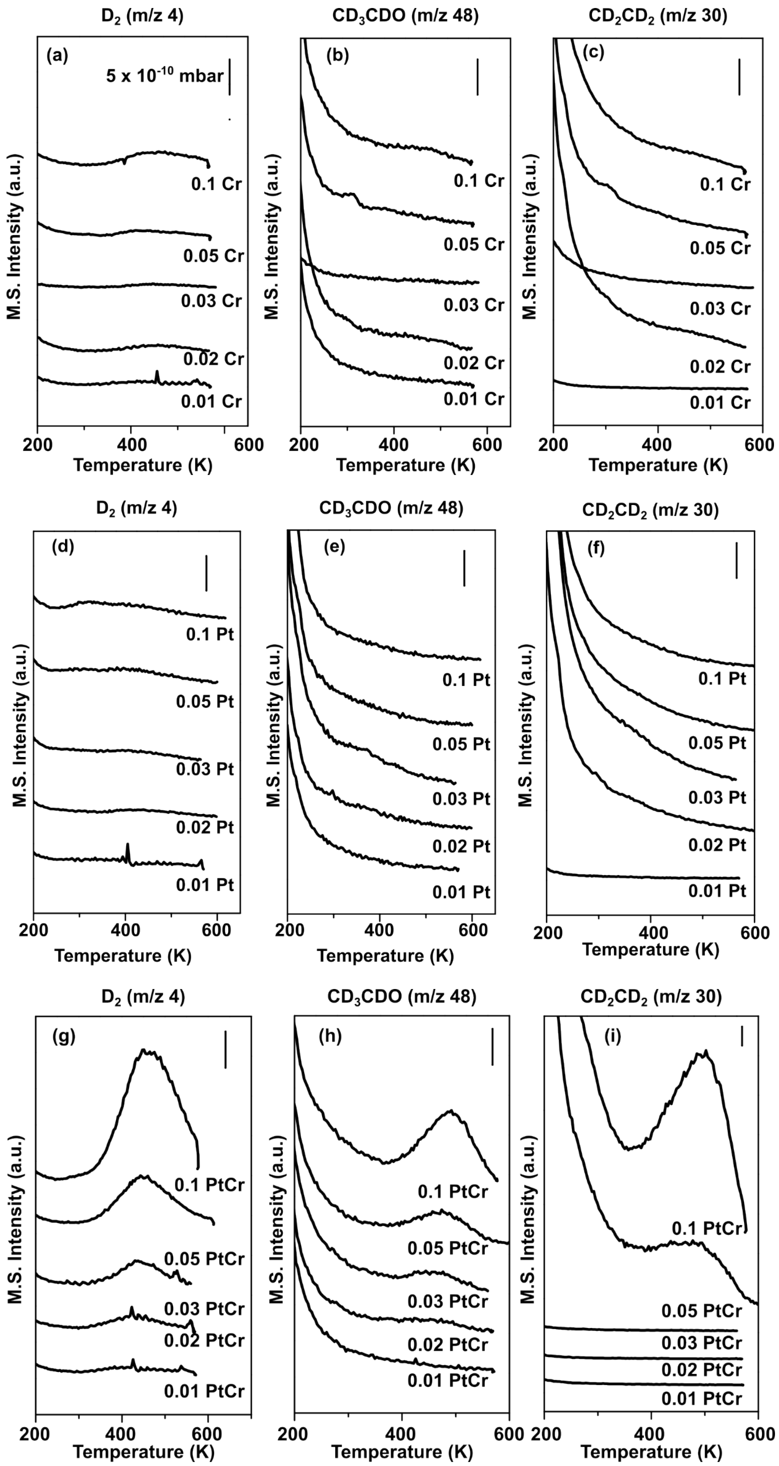

Figure S7 Product yields from the reaction of ethanol on CrAg, PtAg, and CrPtAg surface alloys. Panels a-f demonstrate that both CrAg and PtAg alloys with up to 10% of each dopant are inactive for ethanol decomposition as seen by the lack of acetaldehyde, D_2 and ethylene desorbing. Panels g-i show that when both Pt and Cr are present in the surface, Pt and Cr concentrations as low as 3% lead to the production of D_2 and acetaldehyde, which increases in intensity along with the appearance of ethylene as the dopant coverage increases.

Large Pt aggregates are not expected to react with ethanol based on previous studies which demonstrated reversible molecular adsorption on ethanol on Pt(111).[1] We also performed TPD experiments at a high Cr coverage (0.34 ML) and observed negligible reactivity as compared with the low concentration trimetallic surfaces. Therefore, from Figure S7 it is clear that the low concentration trimetallic surfaces are much more active than the high concentration bimetallic (0.34 Cr) sample, highlighting the synergy between Pt and Cr.

To determine the reaction selectivities, quantitative mass spectrometry was performed after measuring the area under the traces corresponding to ethylene and acetaldehyde. These areas were corrected for ionization cross section, the mass spectrometer quadrupole sensitivity falloff, and fragmentation pattern following the procedure by Siler et al.[1] A value of m/z=48 was chosen for acetaldehyde and m/z=30 for ethylene. The acetaldehyde contribution to m/z=30 was subtracted. After these quantitative corrections, the mass balance closes to within the experimental uncertainty.

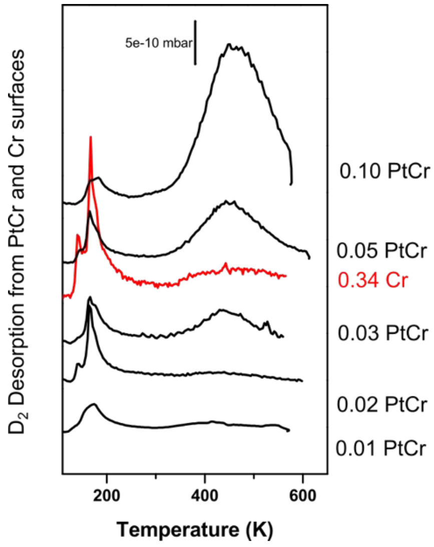

Figure S8. Ethanol reactivity on bimetallic (0.34 ML Cr) vs. low concentration trimetallic PtCrAg samples. D_2 (H_2 for 0.34 Cr case) desorption after ethanol exposure to various alloy surfaces. It is clear that the low concentration trimetallic surfaces are more active than the high concentration bimetallic (0.34 Cr) sample highlighting the synergy between Pt and Cr.

Table S1 Product selectivity from the reaction of 2 L of fully deuterated ethanol (CD_3CD_2OD) with PtCrAg(111) trimetallic alloys as a function of dopant concentration.

Pt:Cr	Ethylene (m/z = 30) Selectivity	Acetaldehyde (m/z = 48) Selectivity
1% : 1%	0%	0%
3% : 3%	0%	100%
5% : 5%	58%	42%
10% : 10%	78%	22%

Table S2: Reaction energies of ethanol dehydrogenation reactions. DFT shows that O-H bond scission favored over C-H bond scission.

Reaction (on Cr_1Pt_1Ag surface)	Reaction Energy / eV	Barrier / eV
$CH_3CH_2OH \rightarrow CH_3CH_2O + H$	-0.15	0.43
$CH_3CH_2OH \rightarrow CH_3CHOH + H$	0.19	No barrier found
$CH_3CH_2OH \rightarrow CH_2CH_2OH + H$	0.37	No barrier found

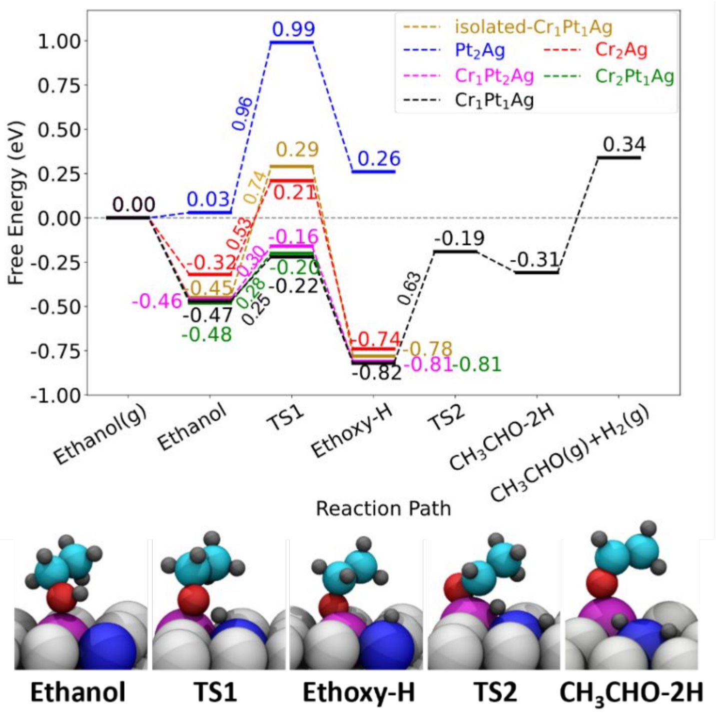

Figure S9 DFT-calculated energetics of ethanol dehydrogenation to acetaldehyde. The heterodimer and trimer sites kinetically and thermodynamically facilitate the reaction steps significantly more than do the homodimer or isolated sites. Free energies calculated at 300 K.

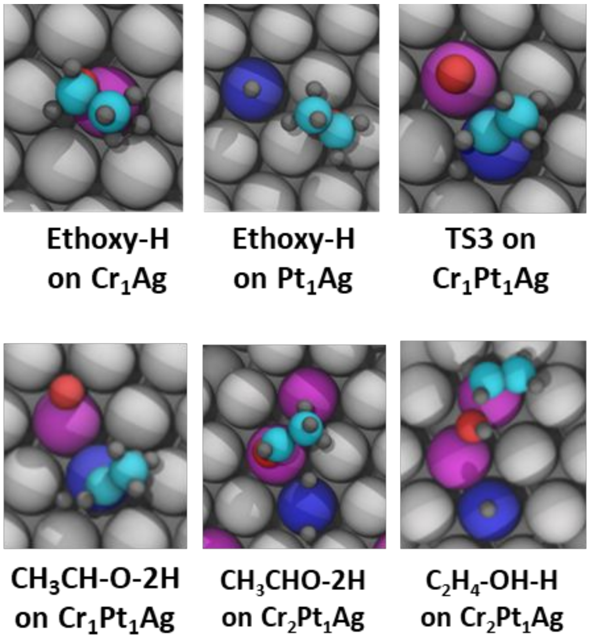

Figure S10. Additional DFT optimized structures in the reaction pathways in Fig. 4. From the acetaldehyde pathway on SAAs: Ethoxy+H on Pt_1Ag and Cr_1Ag. From the ethylene pathway on the Cr_1Pt_1Ag dimer site: TS3 and CH_3CH+O+2H. From the ethylene pathway on the Cr_2Pt_1Ag trimer site: CH_3CHO+2H and C_2H_4+OH+H.

Figure S10 shows additional structures along the reaction pathways for single-atom, dual-atom, and trimer sites. These structures demonstrate that larger ensembles can lead to breaking of more bonds. For example, the dimer site is not large enough to accommodate the multiple fragments leading up to and accompanying the transition state for C-O bond breaking, forcing some intermediates to interact with Ag.

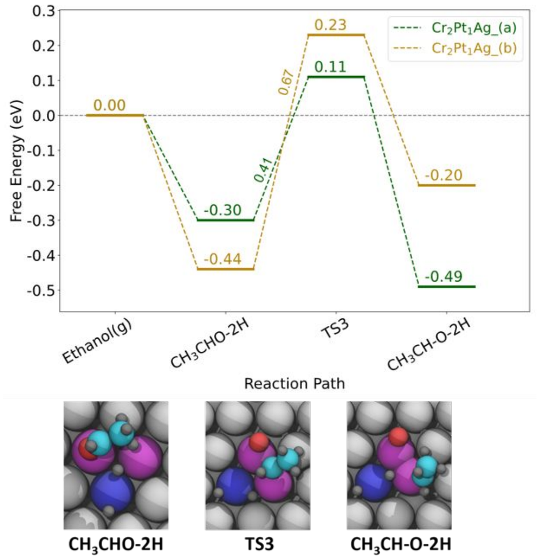

Figure S11. Energetics of breaking C-O bond on two different Cr_2Pt_1Ag surfaces. Cr_2Pt_1Ag_(a) is the surface in the main text, and Cr_2Pt_1Ag_(b) is an alternative trimer structure. Both feature much lower barriers than the Cr_1Pt_1Ag dimer.

We have also calculated CO stretching frequencies on the Pt_1Cr_1Ag pair sites to aid in future experimental characterization of high surface area versions of these materials (see Table S3).

Table S3. DFT-calculated CO stretching frequencies on different sites on Pt_1Cr_1Ag.

CO Position	Stretching frequency (cm^{-1})
Top of Cr	1957
Top of Pt	2000
Bridging Cr and Pt	1793

References

(1) Siler, C. G. F.; Cremer, T.; Rodriguez-Reyes, J. C. F.; Friend, C. M.; Madix, R. J. Switching Selectivity in Oxidation Reactions on Gold: The Mechanism of C–C vs C–H Bond Activation in the Acetate Intermediate on Au(111). *ACS Catal.* **2014**, *4* (9), 3281–3288. https://doi.org/10.1021/cs500803n.
